# ﻿*Impatiens tainingensis* (Balsaminaceae), a new species from southeastern China

**DOI:** 10.3897/phytokeys.269.173115

**Published:** 2026-01-19

**Authors:** Meizhen Wang, Jiedong Lin, Boheng Li, Junlong Li, Xinjie Jin, Pan Li

**Affiliations:** 1 Keqiao Science and Technology Innovation Center, Zhejiang Shuren University, Shaoxing 312028, China Zhejiang Shuren University Shaoxing China; 2 College of Biology and Environmental Engineering, Zhejiang Shuren University, Hangzhou 310015, China Zhejiang Shuren University Hangzhou China; 3 Educational Technology Center of Fujian Province, Fuzhou 350003, China Educational Technology Center of Fujian Province Fuzhou China; 4 YOUNG NATURALIST (Hangzhou) Cultural Communication Co. Ltd., Hangzhou 310058, China YOUNG NATURALIST (Hangzhou) Cultural Communication Co. Ltd. Hangzhou China; 5 College of Life Science, Zhejiang Sci-Tech University, Hangzhou 330104, China Zhejiang Sci-Tech University Hangzhou China; 6 College of Life and Environmental Science, Wenzhou University, Wenzhou 325035, China Wenzhou University Wenzhou China; 7 Key Laboratory of Biodiversity and Environment on the Qinghai-Tibetan Plateau, Ministry of Education, School of Ecology and Environment, Xizang University, Lhasa 850000, China Xizang University Lhasa China; 8 Motuo Biodiversity Observation and Research Station of Xizang Autonomous Region, Motuo 860700, China Motuo Biodiversity Observation and Research Station of Xizang Autonomous Region Motuo China

**Keywords:** Balsaminaceae, *Impatiens kuocangshanica*, *I. tainingensis*, *I. wuyiensis*, new species

## Abstract

*Impatiens tainingensis* J.-D.Lin & P.Li, **sp. nov.**, is described and illustrated. It was collected from a moist valley of Mt. Zhuangyuanyan, Fujian Province, in Southeastern China. It resembles *Impatiens platysepala* Y.L. Chen, *I. kuocangshanica* (X.F. Jin & F.G. Zhang) X.F. Jin & Y.L. Xu and I. huangyanensis subsp. attenuata X.F. Jin & Z.H. Chen in the gross morphology of their pinkish purple flowers but differs in having smaller lateral sepals, subsaccate-funnel form lower sepals, shorter spurs, and capsules. The molecular phylogeny of *Impatiens* based on plastome and ITS sequences indicated that *I. tainingensis* is closely related to *I. platysepala* and *I. wuyiensis* J.S. Wang, Y.F. Lu & X.F. Jin, but morphology, phylogeny, and plastome structural variation comparison provide evidence for recognizing it as a distinct species.

## ﻿Introduction

The family Balsaminaceae consists of two genera, *Hydrocera* Blume ex Wight & Arn. (1834: 140) and *Impatiens* L. (1753: 937). *Hydrocera* is monotypic and contains only the aquatic *H. triflora* (L.) Wight & Arn. (1834: 140), while *Impatiens* has over 1000 species worldwide, mostly distributed in paleotropical and subtropical mountainous regions. As one of the five hotspots for *Impatiens*, China is home to about 352 species, of which 273 are endemic (Yu 2012; Yuan et al. 2022). The mountainous regions in southwestern China, particularly in Yunnan, Sichuan, and Xizang provinces, harbor more than 300 species (Chen et al. 2007; Chen et al. 2023). *Impatiens* is well known for its high level of endemism, with many species confined to very narrow ranges. This pattern is especially pronounced in the karst (limestone) regions of Yunnan, Sichuan, Guizhou, and Guangxi (Chen et al. 2007).

Compared to the species-rich southwestern region of *Impatiens*, fewer species are distributed in the eastern and southern regions (Anhui, Zhejiang, Jiangxi, Fujian, and Guangdong provinces). Nevertheless, the mountainous areas along the Pacific coast in southeastern China, especially the regions associated with the Wuyi Mountain range in Fujian, Jiangxi, and Zhejiang provinces, may represent a secondary diversification center for *Impatiens*. Within this region, Jiangxi hosts 25 species of *Impatiens*, Zhejiang 21 species (six of which are provincial endemics), and Fujian 12 species (including one provincial endemic) (Chen et al. 2023). In addition, 15 taxa have been described in recent years (Chen 1988; Chen 1989; Chen and Xu 1993; Chen 1999; Xu and Chen 1999; Jin and Ding 2002; Jin et al. 2008). It is intriguing that 10 new species were based on plants from Zhejiang. Additional new species might be discovered if extensive botanical investigations are carried out in similar areas with complex topography and rich biodiversity in eastern China, such as Jiangxi and Fujian provinces.

During a survey in Fujian Province, we found a population of *Impatiens* in a valley of Mt. Zhuangyuanyan, Taining County. It appeared to be similar to *I. platysepala* (1993: 6) and *I. kuocangshanica* (2017: 34) in the gross morphology of their pinkish purple flowers, but differed in having smaller lateral sepals, a subsaccate, funnel-formed lower sepal, and shorter spurs and capsules. Molecular phylogeny indicates that it is closely related to *I. platysepala* and *I. wuyiensis* (2020: 3), further confirming the distinct nature of this putative new species. We therefore recognize it as a new species, *Impatiens tainingensis* J.-D.Lin & P.Li, sp. nov., and describe and illustrate it here.

## ﻿Materials and methods

### ﻿Sampling and morphology

**Figure 1.**
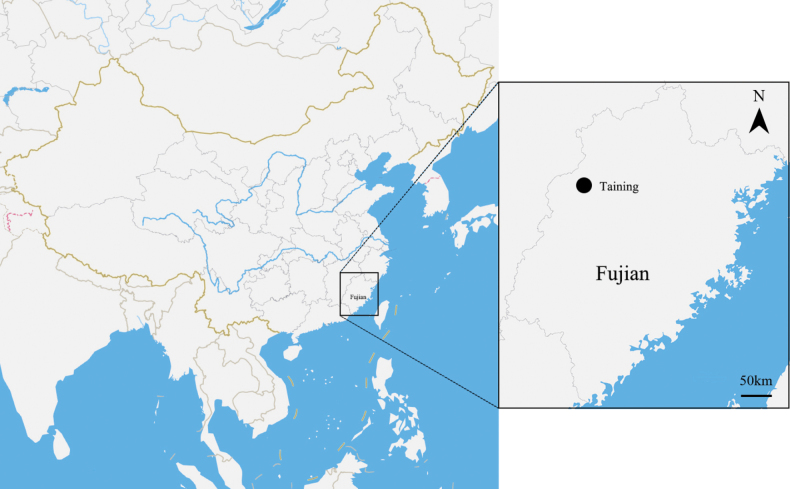
Distribution map of *Impatiens tainingensis*.

We sampled this new species in Taining County, Fujian Province, China (Fig. 1). Morphological characters were observed and measured based on living plants and specimens. Herbarium specimens from AU, BJFC, CSH, CSFI, HHBG, HTC, HZU, IBSC, KUN, LBG, PE, and ZM (acronyms according to Index Herbariorum, https://sweetgum.nybg.org/science/ih/) were examined for morphological comparison. Voucher specimens were deposited at the Herbarium of Zhejiang University (HZU; acronyms according to Index Herbariorum, https://sweetgum.nybg.org/science/ih/).

### ﻿DNA sequencing and phylogenetic analyses

Total genomic DNA was extracted from silica gel–dried leaves using Plant DNAzol (Shanghai, China), following the manufacturer’s protocol. Sequencing libraries were prepared at China National GenBank (CNGB, Shenzhen, China), and paired-end reads of 150 bp were generated on the Illumina HiSeq X10 platform. To determine the phylogenetic relationships of *Impatiens tainingensis*, we retrieved 152 sequences of nuclear ribosomal internal transcribed spacer (ITS), 120 sequences of *atpB–rbcL*, and 70 sequences of *trnL–F* plastid regions from previous *Impatiens* studies (Suppl. material 1; Yu et al. 2016). In addition, 58 complete plastome sequences of *Impatiens* were downloaded from NCBI, and six individuals (two of *I. tainingensis* and one each of I. huangyanensis subsp. attenuata, *I. kuocangshanica*, *I. wuyiensis*, and I. platysepala var. chloroxantha (2017: 34)) were newly sequenced and deposited in GenBank (Suppl. materials 1, 2). Complete plastome and ITS sequences were assembled using GetOrganelle (Jin et al. 2020) with default settings and aligned using MAFFT v7.487 (Katoh and Standley 2013). Maximum likelihood (ML) analyses were performed in IQ-TREE 2 (Minh et al. 2020) with 1000 bootstrap replicates and ModelFinder (Kalyaanamoorthy et al. 2017) for gene partitioning and selection of the best-fit models. Bayesian inference (BI) analyses were conducted in MrBayes v3.2.7a (Ronquist et al. 2012). The Markov chain Monte Carlo (MCMC) algorithm was run with two independent chains and default priors for 10,000,000 generations, with trees sampled every 1000 generations. *Hydrocera triflora* was used as the outgroup. The final tree was visualized and exported using FigTree v1.4.4 (http://tree.bio.ed.ac.uk/software/figtree/).

### ﻿IR/SC boundary comparison

The boundaries of the four plastid regions are LSC/IRb, IRb/SSC, SSC/IRa, and IRa/LSC. The phenomenon of IR region contraction and expansion during plastid evolution in *Impatiens* were analyzed through IR/SC boundary comparisons. The distribution of plastid genes at each boundary was examined using IRscope (https://irscope.shinyapps.io/irapp/, Amiryousefi et al. 2018).

## ﻿Results

### ﻿Morphological comparison

**Figure 2.**
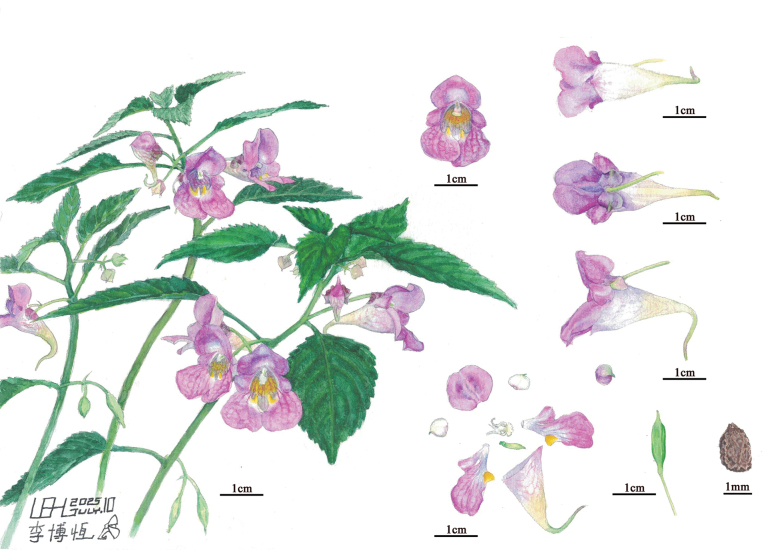
Painted illustration of *Impatiens tainingensis*. (Drawn by Bo-Heng Li).

**Figure 3.**
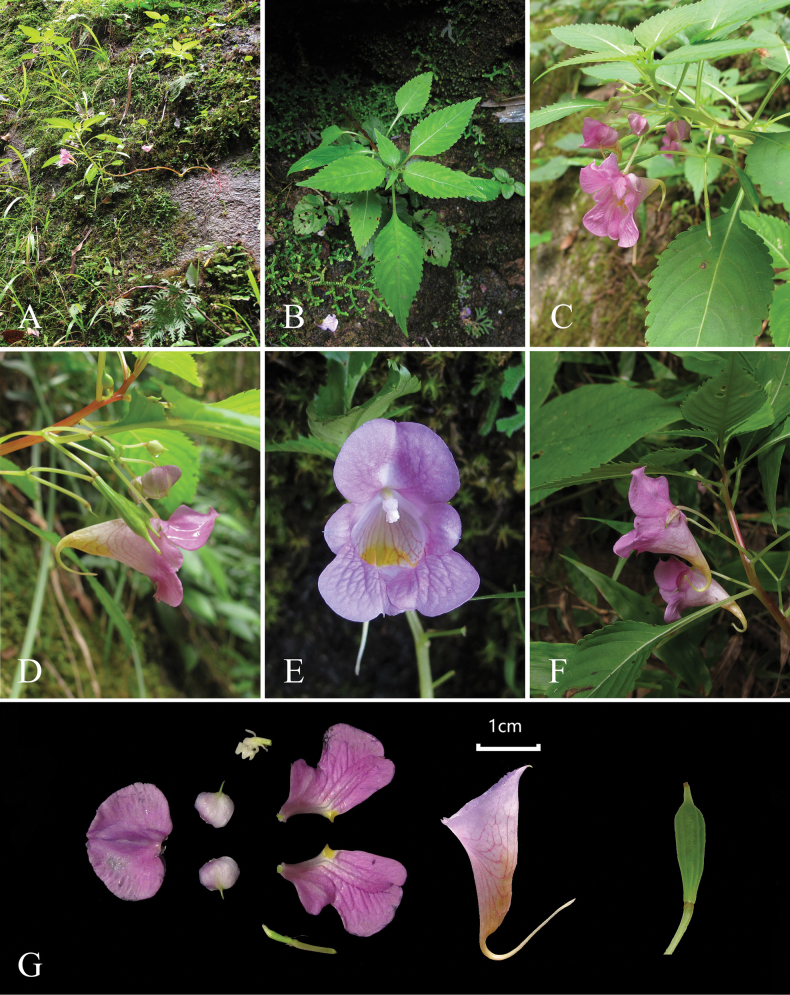
*Impatiens tainingensis*. **A.** Habitat; **B.** Whole plant; **C.** Flower, lateral view; **D.** Inflorescence; **E.** Flower, front view; **F.** Flower, lateral view; **G.** Flower structure and capsule. (Photos by Jie-Dong Lin).

**Figure 4.**
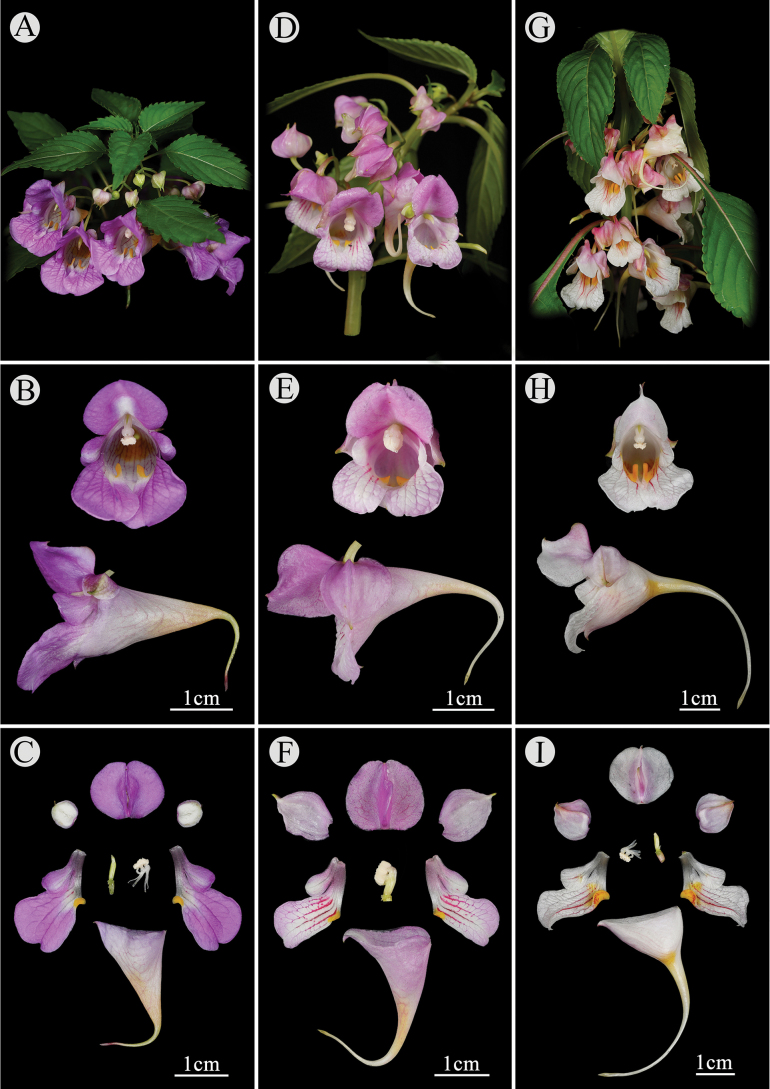
Morphological comparison of *Impatiens tainingensis* (**A–C**), *I. kuocangshanica* (**D–F**), and *I. platysepala* (**G–I**). (Photos by Jun-Long Li).

Detailed morphological comparisons between the new species and three similar *Impatiens* species are summarized in Table 1. *Impatiens tainingensis* (Figs 2, 3) resembles *I. platysepala*, *I. kuocangshanica*, and I. huangyanensis subsp. attenuata in the gross morphology of their pinkish purple flowers but can be distinguished by a combination of leaf and floral characters (Fig. 4). Its leaves are ovate-elliptic with 5–7 pairs of lateral veins, whereas *I. platysepala* has ovate-lanceolate leaves with 9–11 pairs of veins; leaves of *I. kuocangshanica* are similar in size and form to those of *I. tainingensis* but bear stipitate glands (absent in *I. tainingensis*); and I. huangyanensis subsp. attenuata has generally smaller leaves on shorter petioles, although the variation overlaps. In floral morphology, *I. tainingensis* is characterized by small green bracts (larger in *I. platysepala*, *I. kuocangshanica*, and I. huangyanensis subsp. attenuata; pink in *I. platysepala*), suborbicular lateral sepals (broader in *I. platysepala*), and a subsaccate-funnel-formed lower sepal (broadly funnel-formed in *I. kuocangshanica*; widely saccate in I. huangyanensis subsp. attenuata) with a shorter spur (1.5–1.8 cm; 3–4 cm long in *I. platysepala*, *I. kuocangshanica*, and I. huangyanensis subsp. attenuata).

**Table 1.** Comparison between *Impatiens tainingensis*, *I. platysepala*, *I. kuocangshanica*, and *I. huangyanensis* subsp. *attenuate*.

Characters	*I. tainingensis*	*I. platysepala*	*I. kuocangshanica*	I. huangyanensis subsp. attenuata
Plant height	40–80 cm tall	30–50 cm tall	30–50 cm tall	20–40 cm tall
Leaves	petiole 2–4 cm long; leaves ovate-elliptic, 5–11 × 2–3.5 cm, lateral veins 5–7 pairs.	petiole 3–6 cm; leaves ovate-lanceolate, 8–15(-20) × 2.5–5 cm, with stipitate setose glands above middle, lateral veins 9–11 pairs.	petiole 2–6 cm; leaves ovate-elliptic, 5–8.5 × 2.5–4.5 cm, with pairs of stipitate glands, lateral veins 5–9 pairs.	petiole 1–3 cm; leaves ovate-elliptic, 2–6 × 1.5–4 cm, lateral veins 6–8 pairs.
Inflorescences	1–3-flowered; peduncles 1–2 cm. Pedicels 1.5–2.5 cm long; bracts 1–2 mm long, persistent, linear-lanceolate, apex shortly acute, green.	2- or 3-flowered; peduncles 1 cm. Pedicels 1.5–2.5 cm long; bracts 10–12 mm long, ovate-lanceolate, conspicuously cuspidate, pink.	2–4-flowered; peduncles 1 cm. Pedicels 1.5–2.2 cm long; bracts 7–8 mm long, persistent, linear-lanceolate, apex shortly acute, green.	1–3-flowered; peduncles 1 cm long; pedicels 1–2 cm long; bracts 3–4 mm long, persistent, green.
Lateral sepals	suborbicular, 6–8 × 5–8 mm, callous, apex green long mucronulate.	broadly ovate or suborbicular, 10–15 × 10–14 mm, 5-veined, apex shortly cuspidate.	ovate-orbicular, 8–10 × 6–8 mm, apex shortly cuspidate.	ovate rounded, 10–13 × 9–11 mm, apex cuspidate.
Lower sepal	purple striate, 1.9–2.4 cm deep, subsaccate-funnelform, gradually narrowed into an incurved spur; spur 1.5–1.8 cm long; mouth vertical, ca. 1.5–1.7 cm wide.	broadly funnelform, gradually narrowed into a curved or involute spur 3–4 cm; mouth vertical, ca. 2 cm wide, tip acuminate.	broadly funnelform, gradually narrowed into a curved or involute spur 3–4 cm; mouth vertical, ca. 2 cm wide, tip acuminate.	2.0 cm deep, saccate to widely saccate, attenuately constricted to a spur 2–3 cm; mouth vertical, 1.4–1.5 cm wide.
Upper petal	oblate, 1.2–1.3 × 1.7–1.9 cm	suborbicular, 1.6–1.8 cm in diam.	suborbicular, 1.2–1.5 cm in diam.	suborbicular, 1.3 × 1.3 cm
Lateral united petals	basal lobes 9–10 × 6–7 mm, apex obtuse; distal lobes 2.0–2.2 × 1.2–1.4 cm, apex obtuse or emarginate.	basal lobes oblong-obovate, 7–8 mm; distal lobes broadly dolabriform, 1.2–1.3 × 1–1.2 cm, apex obtuse.	basal lobes oblong-obovate, 7–8 mm; distal lobes broadly dolabriform, 1.2–1.3 × 1–1.2 cm, apex obtuse.	basal lobes obovate, 0.7–0.8 × 0.4–0.5 cm; distal lobes 1.7–2.0 × 0.6–0.9 cm
Capsule	fusiform, 1.5–2.0 cm long.	fusiform, 2.5–3.0 cm long.	linear-cylindric, 2.5–3.0 cm long.	linear-cylindric, 2.0–3.0 cm long.
Seeds	2.0 × 1.0 mm	3.0 × 2.0 mm	3.0 × 2.0 mm	2.89 × 1.77 mm

### ﻿Phylogenetic relationship

**Figure 5.**
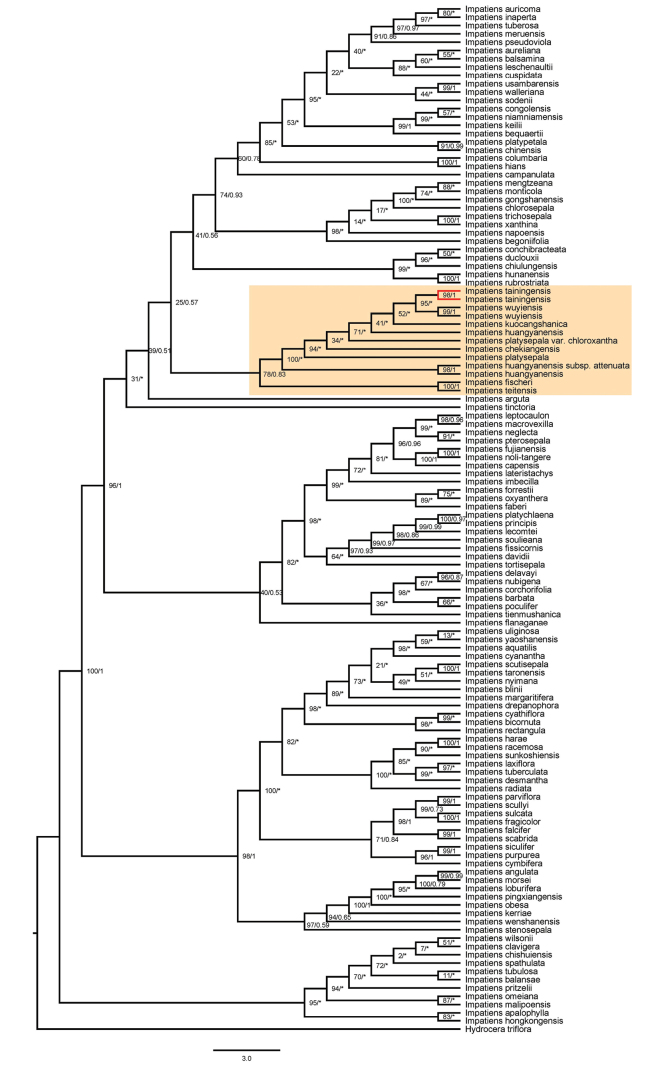
Phylogenetic tree based on *atpB–rbcL* sequences. Numbers opposite nodes indicate bootstrap support percentages and posterior probabilities. An asterisk indicates a comb-like topology or species relationship incongruent with the maximum likelihood tree. *I. tainingensis* is shown in red.

**Figure 6.**
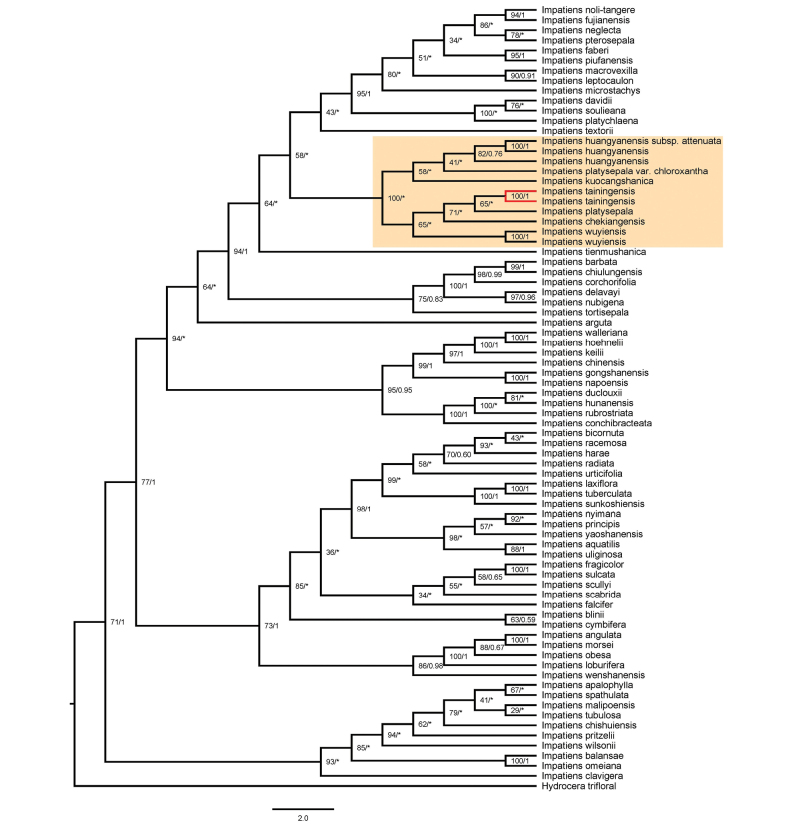
Phylogenetic tree based on *trnL–F* sequences. Numbers opposite nodes indicate bootstrap support percentages and posterior probabilities. An asterisk indicates a comb-like topology or species relationship incongruent with the maximum likelihood tree. *I. tainingensis* is shown in red.

**Figure 7.**
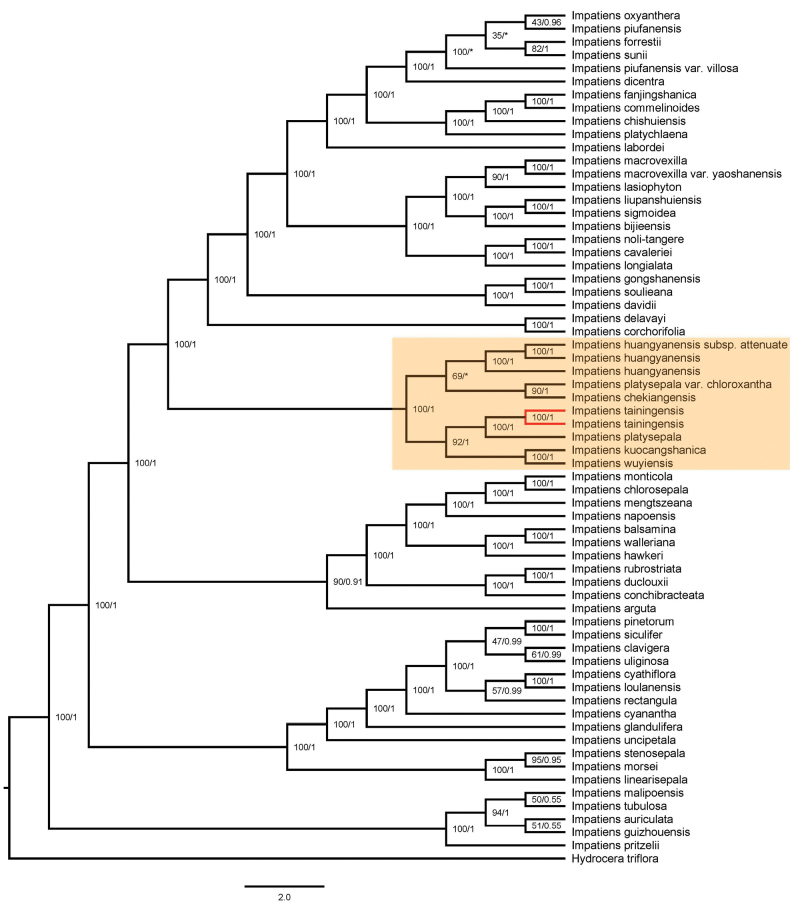
Phylogenetic tree based on complete plastid genome sequences. Numbers opposite nodes indicate bootstrap support percentages and posterior probabilities. An asterisk indicates a comb-like topology or species relationship incongruent with the maximum likelihood tree. *I. tainingensis* is shown in red.

**Figure 8.**
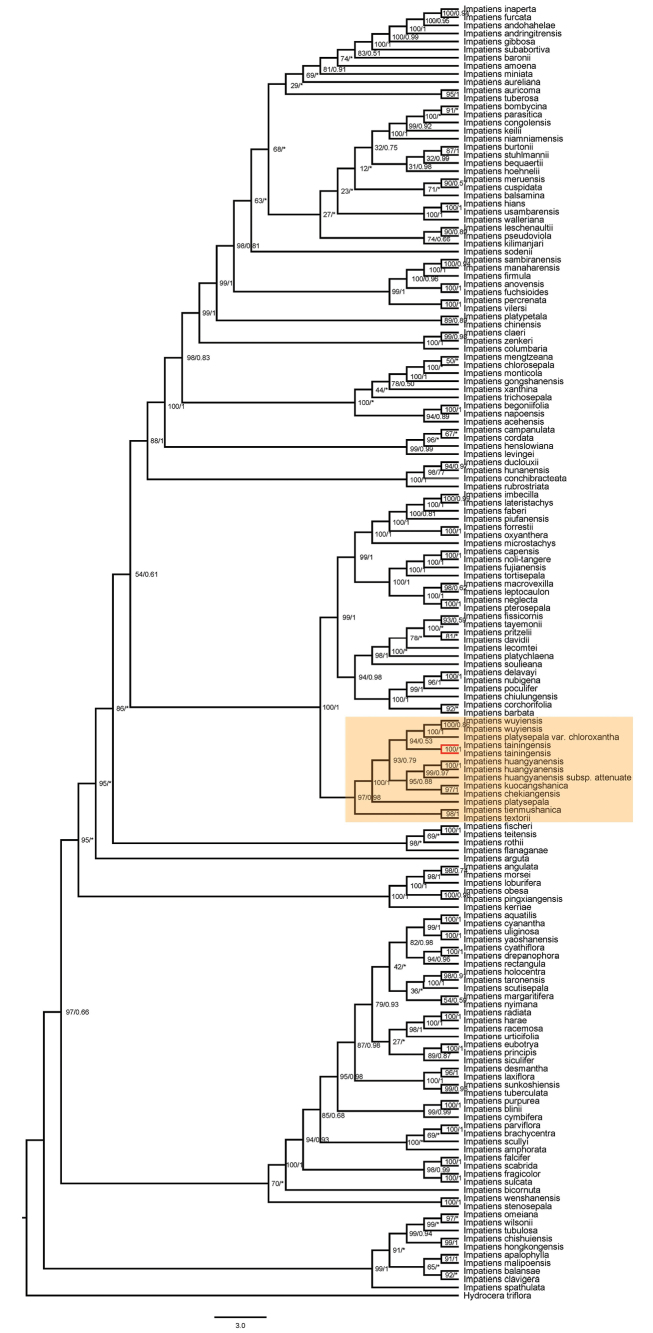
Phylogenetic tree based on ITS sequences. Numbers opposite nodes indicate bootstrap support percentages and posterior probabilities. An asterisk indicates a comb-like topology or species relationship incongruent with the maximum likelihood tree. *I. tainingensis* is shown in red.

The phylogenetic results based on the same dataset inferred using maximum likelihood (ML) and Bayesian inference (BI) methods were consistent for the clade of *Impatiens tainingensis* and related species. Phylogenetic trees based on plastid and internal transcribed spacer (ITS) sequences show a close relationship among *I. tainingensis*, *I. wuyiensis*, and *I. platysepala* (Figs 5–8). The bootstrap support (BS) values and posterior probabilities (PP) for clades including *I. tainingensis* and related species based on *atpB–rbcL* and *trnL–F* sequences were relatively lower than those of the other two datasets (Figs 5, 6). Although *I. tainingensis* was strongly supported as monophyletic, interspecific relationships among closely related taxa remained unresolved, resulting in a comb-like topology in the BI trees (Figs 5, 6). These results are nevertheless largely consistent with phylogenetic relationships inferred from analyses of the complete plastid genome and the ITS dataset. Based on the complete plastid genome, *I. tainingensis* clustered with *I. platysepala*, *I. wuyiensis*, and *I. kuocangshanica* as a highly supported clade (BS = 100, PP = 0.85) (Fig. 7). Based on the ITS sequence, *I. tainingensis* clustered with I. platysepala var. chloroxantha and *I. wuyiensis* as a highly supported clade (BS = 93, PP = 0.79) (Fig. 8).

### ﻿IR/SC boundary comparison

**Figure 9.**
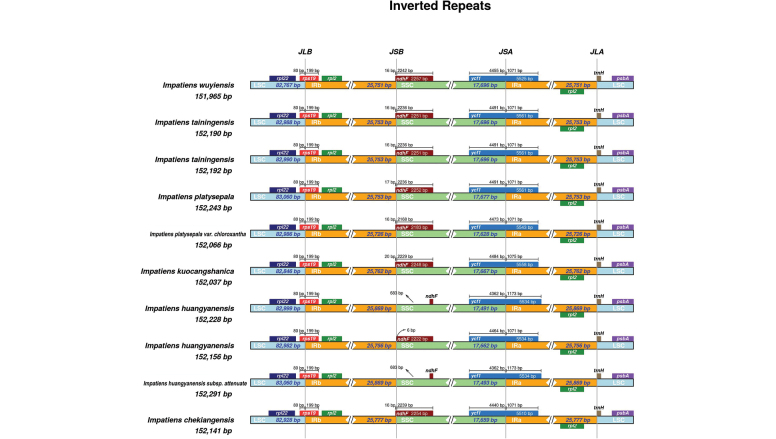
Comparison of IR/SC boundaries among *Impatiens tainingensis* and related species. Positions of genes at the junctions between the large single-copy (LSC), small single-copy (SSC), and inverted repeat regions (IRa and IRb) are shown. Numbers above or within genes indicate lengths (bp) of gene fragments extending across the boundaries. Gene orientations are indicated by arrows. Total plastome sizes for each species are shown on the left.

The results show that the LSC/IRb boundary is located within the *rps19* gene and the IRa/LSC boundary within the *trnH* gene, a pattern that is consistent across all species examined (Fig. 9). Significant differences were observed in the distribution of the *ycf1* and *ndhF* genes at the IR/SC boundary among *Impatiens* species. In *I. huangyanensis*, the *ndhF* gene is entirely located within the SSC region, whereas in the other species examined, 16–17 bp of the gene extend into the IRb region. The *ycf1* gene consistently spans the SSC/IRa boundary, but the length of the portion located within the SSC region varies among species. The plastome IR/SC boundary of *I. tainingensis* is similar to that of *I. platysepala* but differs in the lengths of the IR/SC regions and in the distribution length of the *ndhF* and *ycf1* genes at the boundary (Fig. 9).

### ﻿Taxonomic treatment

#### ﻿ Impatiens tainingensis

Taxon classificationPlantae

J.-D.Lin & P.Li sp. nov.

3E4EC8E4-DF71-5B51-B0C9-DCBC6AF2BCA3

urn:lsid:ipni.org:names:77375257-1

Figs 2, 3

##### Type.

China. Fujian • Taining County, Mt. Zhuangyuanyan, 26°58'12.35"N, 117°11'55.89"E, 312 m a.s.l., 31 Jul 2022, *Jin et al. Jin Xin Jie 349* (holotype: ZM; isotype: HZU, FUN).

##### Diagnosis.

*Impatiens tainingensis* is similar to *I. platysepala*, *I. kuocangshanica* and I. huangyanensis subsp. attenuata in pinkish purple flowers. But *I. tainingensis* differs from *I. platysepala* by its bracts green, persistent, linear-lanceolate, 1–2 mm long; lower sepal subsaccate-funnelform, spur 1.5–1.8 cm long. *I. tainingensis* is distinct from *I. kuocangshanica* and I. huangyanensis subsp. attenuata in having lower sepal subsaccate-funnelform, spur 1.5–1.8 cm long; lateral sepals suborbicular, callous, 6–8 × 5–8 mm; capsules fusiform, 1.5–2.0 cm long and seeds 2.0 × 1.0 mm.

##### Description.

Annual herb, 40–80 cm tall, glabrous. ***Root*** fibrous. ***Stem*** erect or ascending in lower part, slender and fleshy, simple, or laxly branched. ***Leaves*** alternate; petiole 2–4 cm long; blade papery, pale green, ovate-elliptic, 5–11 × 2–3.5 cm, lateral veins 5–7 pairs, curved, base cuneate, attenuate into petiole, margin crenate-serrate or crenate, teeth mucronulate, apex caudate-acuminate; ***Inflorescences*** axillary, 1–3-flowered; peduncles 1–2 cm long. Pedicels 1.5–2.5 cm long, bracteate at base; bracts persistent, linear-lanceolate, 1–2 mm long, apex shortly acute. ***Flowers*** pink-purple, ca. 3 cm deep. Lateral sepals 2, suborbicular, 6–8 × 5–8 mm, callous, margin entire, apex green long mucronulate, abaxial midvein narrowly carinate. Lower sepal purple striate, 1.9–2.4 cm deep, subsaccate-funnelform, gradually narrowed into an incurved spur; spur 1.5–1.8 cm long; mouth vertical, ca. 1.5–1.7 cm wide. Upper petal oblate, 1.2–1.3 × 1.7–1.9 cm, apex emarginate, shortly rostellate, abaxial midvein cristate; lateral united petals, shortly petiolate, 2-lobed; basal lobes obovate to dolabriform, small, 0.9–1.0 × 0.6–0.7 cm, apex obtuse; distal lobes dolabriform, large, 2.0–2.2 × 1.2–1.4 cm, apex obtuse or emarginate; auricle inflexed, yellow; Filaments linear, anthers ovoid, apex obtuse. Ovary erect, fusiform, ca. 5 mm long. ***Capsule*** fusiform or linear-cylindric, 1.5–2.0 cm long; seeds many, yellowish brown, ovoid-oblong, 2.0 × 1.0 mm, tuberculate.

##### Phenology.

Flowering and fruiting from May to September.

##### Distribution and habitat.

Taining, Fujian, southeastern China; known only from the type of location (Fig. 1). Moist places in valleys of Danxia landform; 300–400 m.

##### Conservation Status.

Based on its limited area and extent of occurrence, *Impatiens tainingensis* could be categorized as near threatened (NT) according to IUCN criteria (IUCN Standards and Petitions Committee 2022). It is found at only three sites in Taining County in Fujian Province; there is no obvious downward trend in the population size, and there are currently thousands of individuals at each site.

##### Additional specimens examined (paratypes).

China. Fujian Province • Taining County, Mt. Zhuangyuanyan, 26°58'12.35"N, 117°11'55.89"E, 312 m, 9 August 2014 (fl.), *Jiedong Lin* Jin Xin Jie 397 (HZU).

##### Comparative material examined (allied species).

***Impatiens chekiangensis*** — China. **Fujian** Province • Mt. Wuyi, 400 m, 22 Octorber 1965 (fl.), *Guozong Mao 10513* (HHBG). **Zhejiang** Province • Hangzhou City, Lin’an District, Changhua Town, 960 m, 19 June 1957 (fl. & fr.), *Deng et al. 23509* (HHBG); • Shunxi Town, 280 m, 2 July 1986 (fl.), *Guojiang Zhang L8442165* (IBSC); • Lishui City, Longquan City, 20 July 1958 (fl.), *Renye Sahn 5510* (PE); *ibidem*, 14 September 1959 (fl.), *Shaoyao Zhang 6799* (KUN); • Suichang County, Mt. Jiulong, 400 m, 22 September 1989 (fl.), *Yilin Chen s.n.* (PE); *ibidem*, 850 m, 5 July 1991, *Xu 1115* (ZM); • Zhedaikou Town, Chenkeng Protection Station, 28°19'31"N, 118°49'43"E, 709.46 m, 29 October 2017 (fl. & fr.), *Xin Zhong et al. ZX04178* (CSH); • Taizhou City, Xianju County, Yukeng Nature Reserve, 450 m, 20 July 2000 (fl.), *Xiaofeng Jin 6918* (PE); • Wenzhou City, Yueqing City, Mt Yandang, 250 m, 16 May 2005 (fl.), *Xiaofeng Jin & Peijian Cao 1537* (PE); • Quzhou City, Wuxi River National Wetland Park, 26 April 2019 (fl. & fr.), *Yongfu Xu et al. ZJQZWXJ20190426017* (CSFI); **Jiangxi** Province • Jiujiang City, Wuning County, Songxi Town, Yanxia Village, 29°23'13"N, 115°00'07"E, 145 m, 24 October 2013, *Xuanhuai Zhan et al. LXP0609* (LBG).

***Impatiens fujianensis*** — China. **Fujian** Province • Wuyishan City, Sangu Town, Wuyi Mountain National Park, 27°44'56"N, 117°38'23"E, 1327 m, 4 Octorber 2023 (fl.), *Liang Ma 20231004001* (PE).

***Impatiens huangyanensis*** — China. **Zhejiang** Province • Taizhou City, HuangYan County, Shabu Town, Ersiheng Village, 300 m, 2 August 2000 (fl.), *Xiaofeng Jin 6928* (PE); *ibidem*, 330 m, 2 August 2000 (fl.), *Xiaofeng Jin 6929* (PE).

***Impatiens kuocangshanica*** — China. **Zhejiang** Province • Taizhou City, Xianju County, 15 May 1960 (fl.), *7887* (HHBG); • Yukeng Forest area, 350 m, 26 November 1982 (fl.), *Hangzhou Botanical Garden Herbarium 2788* (HHBG).

***Impatiens platysepala*** — China • **Fujian** Province: fl. & fr., *H.C. Chao 1788* (PE); 20 September 1932 (fl. & fr.), *Daxuan Wang 838* (PE); • Chongan County, Mt. Wuyi, 380 m, 4 July 1974 (fl.), *Team 236-6* 499 (PE); • Nanping City, Songxi County, 27°26'43"N, 118°46'7"E, 675 m, 28 August 2017 (fl.), *Xiangxiu Su CSH22213* (CSH). **Zhejiang** Province • Lishui City, Jingning County, 20 October 1964 (fl.), *Zongguo Mao 10349* (HHBG); • Qingyuan County, Shangzhuang Village, 200 m, 21 September 1989 (fl.), *Yilin Chen 89-02* (PE); • Wenzhou City, Taishun County, Chenmeikeng, 610 m, 5 May 1990 (fl.), *Li et al. 0706* (ZM); • Quzhou City, Jiangshan City, Jianglang Mountain National Scenic Area, 28°31'57"N, 118°33'53"E, 25 April 2017 (fl.), *Xin zhong et al. ZX03052* (CSH); • Taizhou City, Linhai County, Mt. Kuocangshan, 100 m, 2 July 2005 (fl.), *Xiaofeng Jin 1551* (HTC). **Anhui** Province: • Lu’an City, Shucheng County, 500 m, 20 September 1996 (fl.), *M.B. Deng 93034* (PE); • Huangshan City, Xiuning County, Mt. Qiyun, 480 m, 29°48'38"N, 118°1'33"E, 6 June 2012 (fl.), *D.E. Boufford et al. 42916* (PE). **Jiangxi** Province • Shangrao City, Guangfeng District, 24 October 1958 (fl. & fr.), *Minxiang Nie & Shunshen Lai 5930* (PE, KUN); • Wufushan Town, 1000 m, 14 September 1958 (fl.), *Shusheng Lai & Minxiang Nie 5050* (IBSC, KUN); • Gangboshan Nature Reserve, 28°08'22"N, 118°16'42"E, 515 m, 14 June 2018 (fl.), *Zhenji Li et al. AU02680* (AU); • Hengfeng County, Mt. Zheting, 60 m, 23 April 2013 (fl.), *Jianjun Zhou & Dasong Zhou 20130424* (CSFI); • Qianshan County, Wangcun Village, 14 October 2017 (fl. & fr.), *Binjie Ge et al. CSH21867* (CSH).

**Impatiens platysepala var. chloroxantha** — China. **Fujian** Province • Mt. Wuyi, 27°38'20"N, 117°57'7"E, 243 m, 1 July 2019 (fl.), *Xiaohui Song 2936* (AU); • 3 May 2015(fl.), *Xiangxiu Su CFH09011332* (CSH). **Zhejiang** Provinc • Lishui City, Suichang County, Mt. Jiulong, 600 m, 25 September 1989 (fl. & fr.), *Yilin Chen 89-03* (PE); *ibidem*, 550 m, 21 August 1991, *Xu 1234* (ZM); *ibidem*, 751.39 m, 5 June 2012 (fl.), *Qi Tian et al. TQ01758* (CSH); • Hangzhou City, Lin’an District, Mt. Longtang, 850 m, 27 June 1981 (fl.), *Chunsheng Xiang 0020* (HTC); **Hubei** Province • Shennongjia National Park, QianjiaPing, 6 August 2010 (fl.), *Jinyu Li et al. SN0079* (BJFC).

***Impatiens wuyiensis*** — China. **Fujian** Province • Wuyishan City, Yangzhuang Town, Longguiyuan Scenic Area, 27°51'07"N, 117°53'05"E, 519.9 m, 18 July 2021 (fl.), *Jingyi Wang 136* (AU); *ibidem*, 27°51'10"N, 117°53'14"E, 527 m, 18 July 2021 (fl.), *Tao Wei 993* (AU).

### ﻿Key to *Impatiens tainingensis* and related species

**Table:** 

1	Inflorescences subdivaricate, 2(or 3)-flowered; peduncles longer than pedicels	***I. chekiangensis***
–	Inflorescences subumbellate, 2- or 4-flowered; peduncles shorter than pedicels	**2**
2	Bracts thinly membranous, pink, or yellow-green, ovate-lanceolate, 1–1.2 cm long	***I. platysepala* (I. platysepala var. chloroxantha)**
–	Bracts herbaceous, green, linear-lanceolate, shorter than 1 cm	**3**
3	Flower Golden yellow; lateral sepals densely purple-red spotted	***I. wuyiensis***
–	Flower pink-purple; lateral sepals not spotted	**4**
4	Lower sepal subsaccate-funnelform, spur 1.5–1.8 cm long; lateral sepals suborbicular, callous, 6–8 × 5–8 mm	***I. tainingensis***
–	Lower sepal broadly funnelform or saccate, spur 2–4 cm long; lateral sepals ovate-orbicular or ovate rounded, membranous, 8–10 × 6–8 mm or more	**5**
5	Lower sepal broadly funnelform, gradually narrowed into a curved or involute spur 3–4 cm long	***I. kuocangshanica***
–	Lower sepal saccate, base abruptly or gradually narrowed to a spur ca. 2 cm long	***I. huangyanensis* (I. huangyanensis subsp. attenuata)**

## ﻿Discussion

Our integrative morphological and molecular evidence strongly supports the recognition of *Impatiens tainingensis* as an independent species. *Impatiens tainingensis* is distinguishable by a suite of stable morphological features, including notably smaller lateral sepals, a subsaccate-funnel-formed lower sepal, a short spur, and smaller capsules. These floral traits, together with leaf characters such as shorter petioles and fewer lateral veins, provide robust diagnostic characters for precise taxonomic delimitation. Although *I. tainingensis* is morphologically similar to *I. kuocangshanica* and I. huangyanensis subsp. attenuata, these taxa are phylogenetically distant. Phylogenetic analyses based on plastid and internal transcribed spacer (ITS) datasets consistently recovered *I. tainingensis* as a strongly supported monophyletic lineage. Despite *I. tainingensis* being phylogenetically close to *I. wuyiensis* and *I. platysepala* and its subspecific taxa, it differs from *I. platysepala* by its green, persistent, linear-lanceolate bracts (1–2 mm long). Furthermore, I. platysepala var. chloroxantha and *I. wuyiensis* are readily distinguished by their distinctive flower colors, yellow-green and golden-yellow, respectively. Incongruences between plastid and ITS phylogenetic trees may reflect incomplete lineage sorting, hybridization, or heterogeneous gene flow within *Impatiens* (Liang et al. 2025), including species related to *I. tainingensis*, which would be worthy of further investigation.

Comparative plastome analyses focusing on IR/SC boundaries provide additional structural evidence for the distinctiveness of *I. tainingensis*. Although its IR/SC junction is generally similar to that of *I. platysepala*, *I. tainingensis* exhibits differences in the lengths of IR and SC regions, as well as in the distribution patterns of the *ndhF* and *ycf1* genes across IR/SSC boundaries. Although these plastome variations alone are insufficient to define species boundaries, independent genome-structural characters—such as IR boundary shifts, repeat dynamics, and mutational hotspot divergence—when integrated with phylogenetic and morphological evidence, can provide complementary information and refine taxonomic resolution in morphologically complex groups (Luo et al. 2021; Qiu et al. 2023).

In summary, the integration of morphological distinctiveness, molecular phylogenetic evidence, and plastome structural variation provides strong support for the recognition of *Impatiens tainingensis* as a new species.

## Supplementary Material

XML Treatment for Impatiens tainingensis

## ﻿Additional information

### Use of AI

No use of AI was reported.

### Author ORCIDs

Meizhen Wang https://orcid.org/0000-0002-3794-465X

Jiedong Lin https://orcid.org/0000-0002-2966-1561

Boheng Li https://orcid.org/0009-0005-8847-6883

Junlong Li https://orcid.org/0009-0004-7694-032X

Xinjie Jin https://orcid.org/0000-0003-3733-1957

Pan Li https://orcid.org/0000-0002-9407-7740

### Data availability

All of the data that support the findings of this study are available in the main text or Supplementary Information.

## Supplementary materials

Supplementary material 1 GenBank accession numbers Data type csv Explanation note Species and GenBank accession numbers for the sequences used in this study. https://binary.pensoft.net/file/1515096This dataset is made available under the Open Database License (http://opendatacommons.org/licenses/odbl/1.0/). The Open Database License (ODbL) is a license agreement intended to allow users to freely share, modify, and use this Dataset while maintaining this same freedom for others, provided that the original source and author(s) are credited.Meizhen Wang, Jiedong Lin, Boheng Li, Junlong Li, Xinjie Jin, Pan Li

Supplementary material 2 Collection information Data type csv Explanation note Collection information for newly sequenced samples. https://binary.pensoft.net/file/1515097This dataset is made available under the Open Database License (http://opendatacommons.org/licenses/odbl/1.0/). The Open Database License (ODbL) is a license agreement intended to allow users to freely share, modify, and use this Dataset while maintaining this same freedom for others, provided that the original source and author(s) are credited.Meizhen Wang, Jiedong Lin, Boheng Li, Junlong Li, Xinjie Jin, Pan Li
